# Notes on Ethyl Chloride as a General Anæsthetic
^1^Read before the Bristol Medico-Chirurgical Society, 11th Dec., 1901.


**Published:** 1902-03

**Authors:** Frank H. Rose

**Affiliations:** Anæsthetist to the Bristol Royal Hospital for Sick Children and Women


### NOTES ON ETHYL CHLORIDE AS A GENERAL ANÆSTHETIC.[1]

BY

FRANK H. ROSE, M.R.C.S., L.R.C.P. (Lond.),

*Anæsthetist to the Bristol Royal Hospital for Sick Children and Women.*

HAVING administered or been present at the administration of ethyl chloride in over one hundred cases, I thought that some particulars of the anæsthetic as recorded by others, and noticed by myself, might be of interest.

My own experience of it is in operations about the mouth and throat, in dental work, and for the removal of tonsils and adenoids, and therefore it is only of short anæsthesia that I speak. But it has been successfully administered in operations of eighteen and thirty minutes duration, and in one of fifty minutes for the removal of tubercular testis.

For some years ethyl chloride has been used to induce local anæsthesia, and in 1895 a dentist at Gothenburg observed that occasionally, when used for its local effect only, in teeth extraction, a general narcosis supervened, from which the patient awoke after the extraction declaring that he had felt neither pain nor inconvenience. This led to further investigations, with the result that more or less lengthy operations have been performed by its aid, as well as some thousands of short ones in which nitrous oxide might have been used.

[1] Read before the Bristol Medico-Chirurgical Society, 11th Dec., 1901.

The points in favour of ethyl chloride are:—1st. Its safety. The pulse and respiration are practically unaffected; though the former may be slowed slightly, the volume is unaltered. Up to March last, out of a collection of 2,500 cases, by Lotheissen of Innsbruck, only one death is recorded, that of an extremely bad subject. 2nd. Its pleasant odour. 3rd. Easy induction of anæsthesia, averaging from one and a half to two minutes, and in some cases thirty seconds or less. 4th. The extremely rapid and satisfactory recovery from anæsthesia, with practically no disagreeable or troublesome after-effects, and with no irritating action on heart, lungs or kidneys. 5th. A longer period of anæsthesia for short operations in the throat or mouth than is obtained from nitrous oxide—viz., from three-quarters to two minutes; while congestion is not caused, but rather a stimulative action is produced. 6th. Its portability. My first administration for removal of tonsils and adenoids gave a period of operative anæsthesia allowing a digital examination of pharynx, removal of adenoids and both tonsils, and a final clearing of the pharynx with the finger.

The chief disadvantage noticed is that muscular relaxation is not generally complete. Ethyl chloride can be administered to a patient in the recumbent or in the upright sitting position, and special preparation as for ether or chloroform is not necessary; though due care in this respect, as well as in the examination of the heart, is advisable.

As a rule, breathing is fairly regular, except in very neurotic persons, or alcoholics, or young children who are frightened by the mask. I have had one case, a boy of 8 years, where there was a respiratory tonic spasm, lasting from fifteeen to twenty seconds, which was relieved by removing the mask; several cases in which the breathing was at first very irregular; and one case, of a girl, in which there was some jactitation and stertor; as a rule, however, the respiratory difficulties are small and easily relieved. There is practically no cyanosis or pallor, though in some cases I have noticed faint indications in one or other direction.

The pulse is said to be very slightly slowed at the commencement of administration, but I have noticed no variation worth recording.

I have administered ethyl chloride to a woman of 35, with a loud double mitral murmur, for tooth extraction. She has had up to the present four administrations on successive weeks, and has had no trouble or inconvenience whatever; indeed, after the first and second inductions, she appeared to look forward to it with pleasure.

The conjunctival reflex is sometimes lost, but is more frequently dulled only, and the pupil does not invariably contract. At first I found it difficult to know exactly when the patient was fully "under," as sometimes struggling and phonation occur during operation, but I have had less uncertainty in this matter of late. In one case, a strong, vigorous girl of 26 screamed at the top of her voice during the greater part of the time of administration; only calming down when the extraction of two molar teeth was commenced. Immediately after the operation she smiled, and said she knew she had begun to scream, but she didn't know why, as she had felt neither pain nor unpleasant sensations.

The method of administration recommended is by a Breuer's mask. This is somewhat similar to the face-piece of a Clover's ether apparatus, and is fitted with an inspiratory and an expiratory valve: over the former is a metal ball, in which cotton wool is placed; upon this wool 2 or 3 c.c. of ethyl chloride are poured, followed in half a minute by 2 c.c. more, and so on, if necessary, until from 5 to 10 c.c. have been used. For mouth operations, as ethyl chloride is so rapidly eliminated from the system, it is well to have a gag in position before commencing administration.

It is recommended that all air should be carefully excluded, except such as is charged with the vapour; but I have found that anæsthesia is easily and quickly induced when using a lint mask, and I do not see why all exclusion of air is necessary, considering that when ethyl chloride is used for freezing the gums, and narcosis is induced, the vaponr is inhaled freely mixed with air. The first and most marked case in which I tried this method was that of a child of

5 years, who had several carious teeth and alveolar abscesses. The face was bandaged, and the child was evidently in great pain; her breath was so foul that I did not care to use the Breuer's mask, so substituted the ordinary mask which I use for chloroform, merely blocking the mouth-way with lint—the child breathed well, was completely under, and the limbs were flaccid in rather less than one minute. Operative anæsthesia lasted one and a half minutes, during which four teeth were extracted; 4 c.c. of ethyl chloride were used, and the child woke perfectly conscious, and had no unpleasant after-effects. I have now discontinued the use of Breuer's mask, and am having one made to my own design, by which I hope to obviate the freezing which so frequently delays anæsthesia.

I have had two cases of vomiting during administration; in one of them a hearty meal had been taken about two hours before. Where vomiting after operation has occurred, it has been due, I think, to the presence of swallowed blood in the stomach.

Recovery from anæsthesia is very rapid. In cne case, a boy of seven, after extraction of two teeth in the horizontal position, immediately sat up, then jumped off the table and ran out of the room.

In almost all cases the patient speedily regains consciousness, and very rarely complains of having felt pain, except in cases where the operation has clearly exceeded the period of anæsthesia; although struggling during operation is not infrequent.

In conclusion, I consider that ethyl chloride compares favourably with nitrous oxide, in that the period of anæsthesia is longer, and that the condition of analgesia is more marked. An insufficient dose of nitrous oxide allows a patient to feel the pain of an operation, although he may be unable to complain; whereas with ethyl chloride, a patient apparently is conscious of no pain, though he may struggle and phonate.

With an improved mask, and an increased knowledge of the drug and its administration, combined with the portability of the apparatus, etc., I think we have in ethyl chloride an extremely safe and useful anæsthetic.

